# Conditional inactivation of the ectodomain shedding of pro-TNFα in monocytes prevents lethality from LPS-induced septic shock

**DOI:** 10.1186/ar3626

**Published:** 2012-02-09

**Authors:** Keisuke Horiuchi, Tokuhiro Kimura, Yasunori Okada, Kazuhiro Chiba, Carl P Blobel, Yoshiaki Toyama

**Affiliations:** 1 Department of Orthopedic Surgery, School of Medicine, Keio Univ. Tokyo, Japan; 2 Department of Pathology, School of Medicine, Keio Univ. Tokyo, Japan; 3 Arthritis and Tissue Degeneration Program, Hospital for Special Surgery, New York, USA

## Background

TNFα is synthesized as a membrane-bound precursor and proteolytically released from cells. Soluble TNFα is the primary mediator of pathologies such as rheumatoid arthritis, Crohn's disease, and endotoxin shock. Although several different enzymes have been implicated in this proteolytic activity, recent studies lean toward the TNFα converting enzyme (TACE/ADAM17) as the most relevant TNFαsheddase* in *vivo. In the present study, we asked whether the inactivation TACE could yield a protection from lipopolysaccharide(LPS)-induced septic shockin mice.

## Materials and methods

To abrogate TNFα shedding activity in vivo, we generated conditional TACE-deficient mice using Cre-loxP system [1]. We mated these mice with *Mx1-Cre*tg mice and *LysM-Cre*tg mice to inactivate TACE in BM cells and macrophage/monocyte lineage cells, respectively. Endotoxin shock was induced by i.p. injection of 5 μg of LPS and 20 mg of D-galactosamine. All injected mice were closely monitored every hour for the first 16 h and every 3-6 h thereafter.

## Results/conclusions

We found that temporal disruption of TACE under the control of *Mx1 *transgene prevented lethality from endotoxin shock. Furthermore, inactivation of TACE in macrophage/monocyte lineage cells also rendered significant protection against LPS-induced septic shock. Consistent with these findings, serum TNFα levels in the TACE mutant mice were much lower than those in control mice. The present study thus shows that 1) TACE is indeed a principal enzyme responsible for the release of soluble TNFα in vivo, and that 2) inactivation of TACE in macrophage/monocyte lineage cells is sufficient to yield strong protection against LPS-induced endotoxin shock. Taken together, the present data indicate inhibition of TACE activity as a potential therapeutic target for TNFα-related disorders.
